# Dystrophic calcinosis cutis in chronic venous disease of the lower limbs

**DOI:** 10.1590/1677-5449.202101661

**Published:** 2022-09-02

**Authors:** Carolina Heil Arostegui Pacheco, Carmen Lucia Lascasas Porto, Juliana de Miranda Vieira, Ana Leticia de Mattos Milhomens, Rossano Kepler Alvim Fiorelli, Stenio Karlos Alvim Fiorell, Marcos Arêas Marques

**Affiliations:** 1 Universidade do Estado do Rio de Janeiro – UERJ, Hospital Universitário Pedro Ernesto – HUPE, Rio de Janeiro, RJ, Brasil.; 2 Universidade do Estado do Rio de Janeiro – UERJ, Rio de Janeiro, RJ, Brasil.; 3 Universidade Federal do Estado do Rio de Janeiro – UNIRIO, Hospital Universitário Gaffrée e Guinle, Rio de Janeiro, RJ, Brasil.; 4 Universidade Federal do Estado do Rio de Janeiro – UNIRIO, Rio de Janeiro, RJ, Brasil.

**Keywords:** venous insufficiency, skin ulcer, leg ulcer, varicose ulcer, calcinosis, wound healing

## Abstract

Lower limb ulcers secondary to chronic venous disease (CVD) are a significant public health problem in Brazil and account for about 70% of these ulcers. Despite recent technological advances and the various therapeutic options for treatment of these chronic injuries, several factors may be involved in resistance to treatment. Dystrophic calcinosis cutis (DCC) is a rare and often underdiagnosed condition that, when in conjunction with CVD, may be associated with a refractory healing process. In this article, we report a case of DCC in a patient with CVD and discuss its etiology, pathophysiology and possible treatment options.

## INTRODUCTION

Calcinosis cutis (CC) is characterized by abnormal deposition of calcium salts in the skin and subcutaneous cellular tissue (SCT) and can be divided into five subtypes: dystrophic, metastatic, iatrogenic, idiopathic, and calciphylaxis.^1^-^3^

Dystrophic calcinosis cutis (DCC) occurs in patients who do not have systemic calcium metabolism dysfunction^3^,^4^ and can be triggered by local damage, such as trauma, infection, or cancer, causing degeneration of collagen and elastic fibers.^5^

While the pathogenesis of DCC in chronic venous disease (CVD) is not yet fully understood, a small series of published cases attributed the deposits of insoluble calcium phosphate in tissues that amplifies and perpetuates the local inflammatory reaction to venous stasis and the chronic cutaneous inflammatory process, which causes difficulties with healing. In addition to CVD, there are also reports of associations between DCC and *diabetes mellitus*, hyperparathyroidism, advanced chronic kidney disease, autoimmune diseases, connective tissue diseases, cancer, and skin infections.^1^,^2^,^5^

This report describes the case of a patient with ulcers of the left lower limb secondary to CVD, with onset around 24 years previously and refractory to treatment because of concurrent DCC.

This study was approved by the Research Ethics Committee at the Hospital Universitário Pedro Ernesto (HUPE), Universidade do Estado do Rio de Janeiro (UERJ), consolidated opinion number: 5.174.833 (CAAE: 52753821.2.0000.5259).

## CASE DESCRIPTION

The patient was a 70-year-old male with systemic arterial hypertension, type I *diabetes mellitus*, body mass index exceeding 30 kg/m^2^, and CVD. He reported onset of ulcers on his left lower limb at 46 years of age, with duration of around 20 years and full healing for 1 year within that time span. The patient’s ulcerations had relapsed around 2 years previously, when he was referred to the Hospital Universitário Pedro Ernesto (HUPE), part of the Universidade do Estado do Rio de Janeiro (UERJ).

The patient’s previous medical history included recurrent erysipelas of the lower limbs and ultrasound guided dense foam sclerotherapy of the left great saphenous vein around 2 years previously.

**Figure 1:**
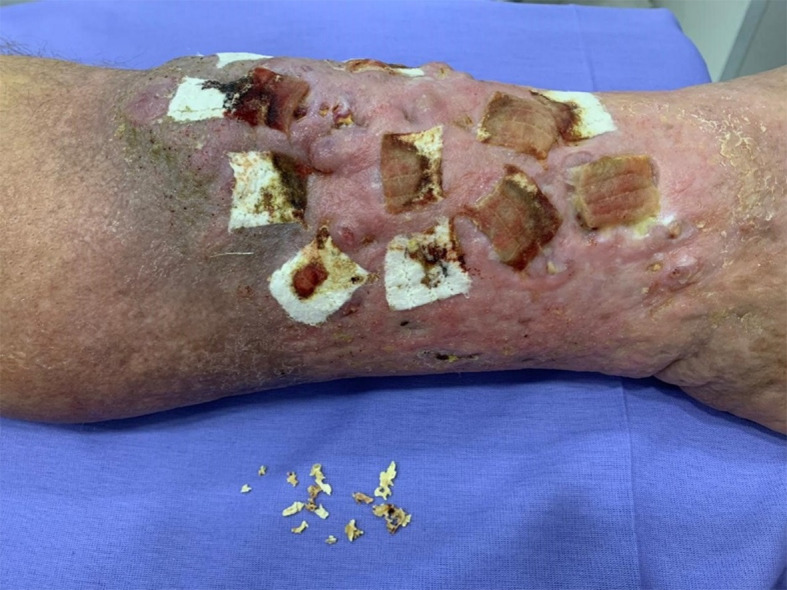
Multiple ulcers on the mid-distal third of the left leg, with diameters varying from 1.0 to 2.5 cm, with moderate exudate and presence of spontaneous extrusion of fragmented, stony, white material, but free from signs of secondary infection. Dressed with calcium alginate and sodium.

Physical examination revealed “boot” hyperpigmentation of the mid and lower thirds of the lower limbs and “orange peel” dry skin with areas of eczema. The patient also had edema 2+/4+, lipodermatosclerosis, lymphatic vesicles, multiple ulcerous lesions of the mid-distal third of the left leg, with diameters varying from 1.0 to 2.5 cm, moderate exudate, no signs of secondary infection, and presence of spontaneous exudate of fragmented, stony, white-colored material (Figure 1). The right lower limb had no ulcers. Lower limb arterial pulses had high-amplitude, equal rhythm and equal pressure.

It was notable that the results of laboratory tests of calcium metabolism parameters were normal (total calcium: 9.8 mg/dL, phosphorus: 4.0 mg/dL, parathormone: 57 pg/mL, serum creatinine: 0.9 mg/dL, and 25-hydroxy vitamin D: 35 ng/mL).

Laboratory tests for investigation of connective tissue diseases were non-reactive and cultures for bacterial and fungal infections were negative. Pathology tests were ordered, showing DCC and diffuse fibrotic angiodermatitis.

**Figure 2:**
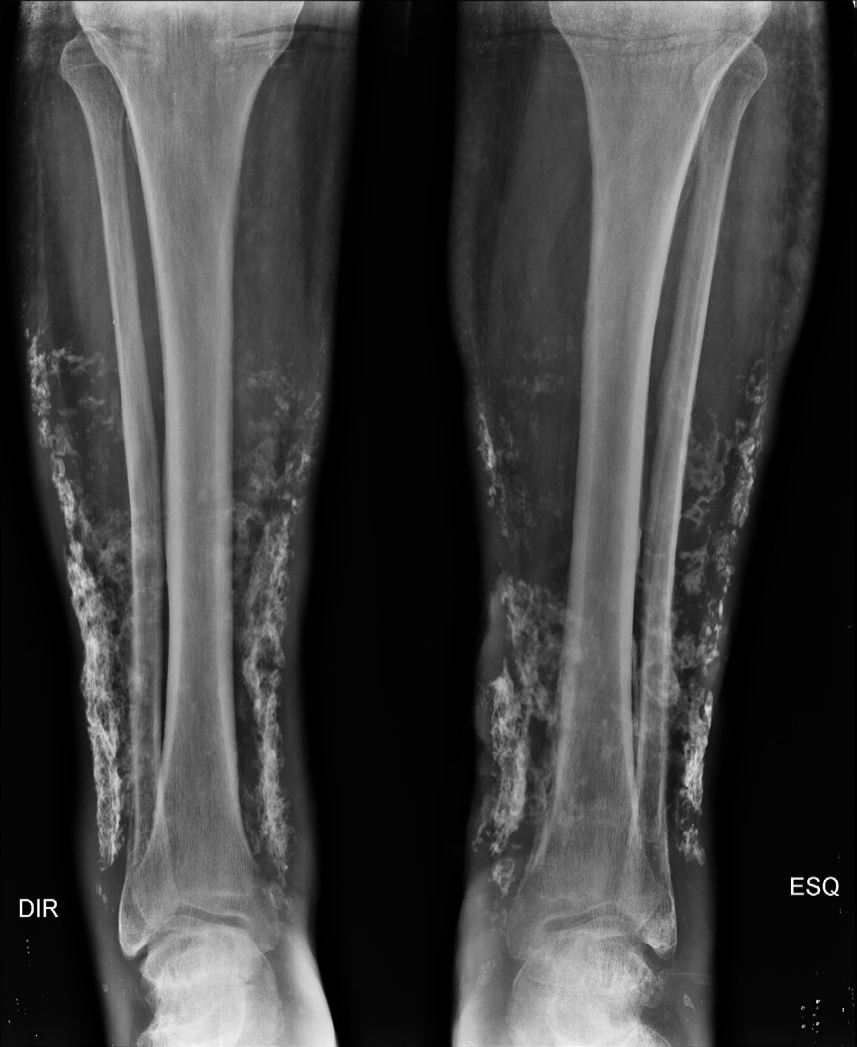
Simple X-rays of the lower limbs: lesions compatible with amorphous calcifications of soft tissues of the external aspect of the mid and lower third of the legs.

Simple X-rays of the left lower limb revealed lesions compatible with amorphous calcifications of soft tissues of the external aspect of the mid and inferior third of the left leg (Figure 2).

**Figure 3:**
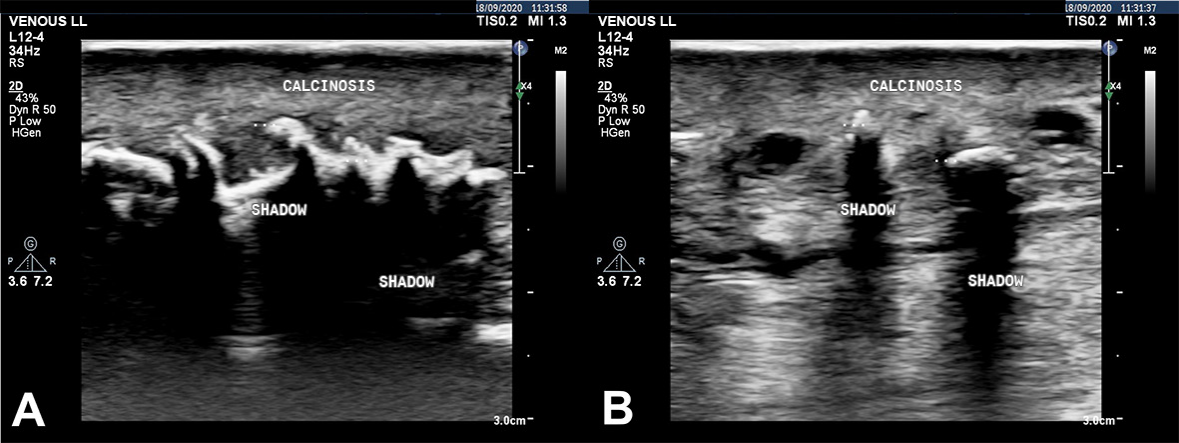
(A) and (B) Color Doppler ultrasound of the lower limbs (B-mode) with hyperechogenic images that generate intense acoustic shadows, with areas of convergence, suggestive of calcium deposits in the subcutaneous cellular tissues, in the mid-distal third of the leg in the region of the ulcers.

Color Doppler ultrasound (CDUS) of the lower limbs revealed reflux in the left great saphenous vein and ruled out signs of acute or previous deep vein thrombosis. During B-mode scanning of the subcutaneous level, hyperechogenic images were observed, generating intense sonic shadows with areas of confluence, suggestive of calcium deposits in the SCT of the distal mid third of the leg, in the region of the ulcers (Figures 3A and 3B).

**Figure 4:**
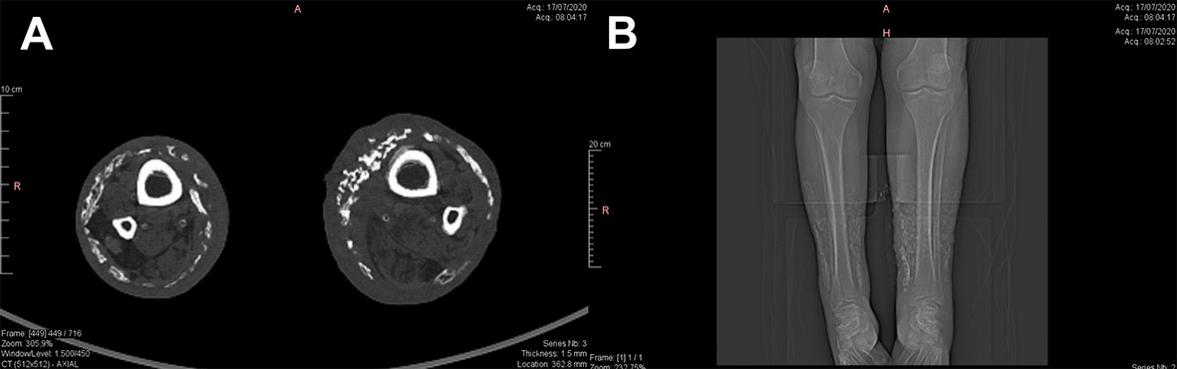
A) Computed tomography of the lower limbs (transverse and longitudinal slices) showing diffuse calcium deposits distributed concentrically around the mid and distal thirds of both lower limbs, with no deposits present in other regions of the body.

Computed tomography of the lower limbs showed diffuse calcium deposits with a concentric distribution in the mid and distal thirds of both lower limbs, with no deposits in other areas of the body (Figures 4A and 4B). The arterial examination showed triphasic flows with normal velocities.

The treatment chosen was a conservative approach with regular dressing applied in the outpatients center, removal of the stony material with forceps, and application of calcium alginate and sodium (Curatec^®^).

## DISCUSSION

Five CC subtypes are described in the medical literature: metastatic, iatrogenic, idiopathic, dystrophic, and calciphylaxis.^1^-^3^,^5^

Metastatic CC involves abnormal calcium and phosphorus metabolism, leading to hypercalcemia and/or hyperphosphatemia; while iatrogenic CC is secondary to intravenous administration of calcium gluconate or treatment with phosphate.^2^,^6^

The etiology of idiopathic CC has not been explained and examples of this condition include scrotal calcification in young men and calcifed nodules in the head or extremities of healthy individuals, without any type of calcium metabolism disorder or damage to underlying tissues.^2^,^5^,^6^

Calciphylaxis is defined as calcification of the middle layer of small and medium blood vessels in the dermis and SCT of patients with end stage chronic kidney disease. Clinically, calciphylaxis can manifest with livedo reticularis racemosa, which progresses to retiform purpura and cutaneous necrosis.^2^,^6^,^7^

Dystrophic calcinosis cutis is related to traumatic, chemical, or infectious injury that leads to abnormal deposition of calcium salts composed of hydroxyapatite and amorphous calcium phosphate in the skin, in patients without systemic calcium metabolism dysfunction. This buildup provokes persistent tissue inflammation which eventually causes skin hypoxia and hypovascularization and induces release of phosphate binding proteins, promoting tissue calcification. Proinflammatory cytokines such as interleukins IL-6 and IL-1 beta and tumor necrosis factor alpha are also related to this process.^2^,^5^,^8^,^9^

In DCC, both metabolism and serum levels of calcium and phosphate are normal and, histology reveals calcium salts in the dermis or subcutaneous fat.^5^ Furthermore, the process of intracellular calcification begins at the mitochondrial level of cells in apoptosis, which lose their capacity to self-regulate calcium levels, permitting its ingress in large quantities and provoking failure of metabolism, necrosis, and cell death, culminating in build up of calcium salts in a form similar to apatite.^2^,^5^

Histology shows calcium salts in the form of fine granules and small deposits in the dermis or amorphous deposits in subcutaneous tissues. Their frequency ranges from 10 to 40% in reports in the literature, depending on the assessment technique used.^10^ Lippmann and Goldin^11^ reported calcification of the SCT in 10% of 60 patients with CVD assessed radiographically. Other studies using simple X-rays found evidence that DCC varies from 17.5% to 40% in patients with CVD.^12^,^13^ However, Takoro et al. 4 reported prevalence exceeding 65% of latent DCC in CVD, compared with the general population, suggesting that early detection of latent calcinosis with simple X-rays could be useful for attempts to inhibit its progression.

One limitation of this study is that the patient was lost to follow-up.

## TREATMENT

**Figure 5:**
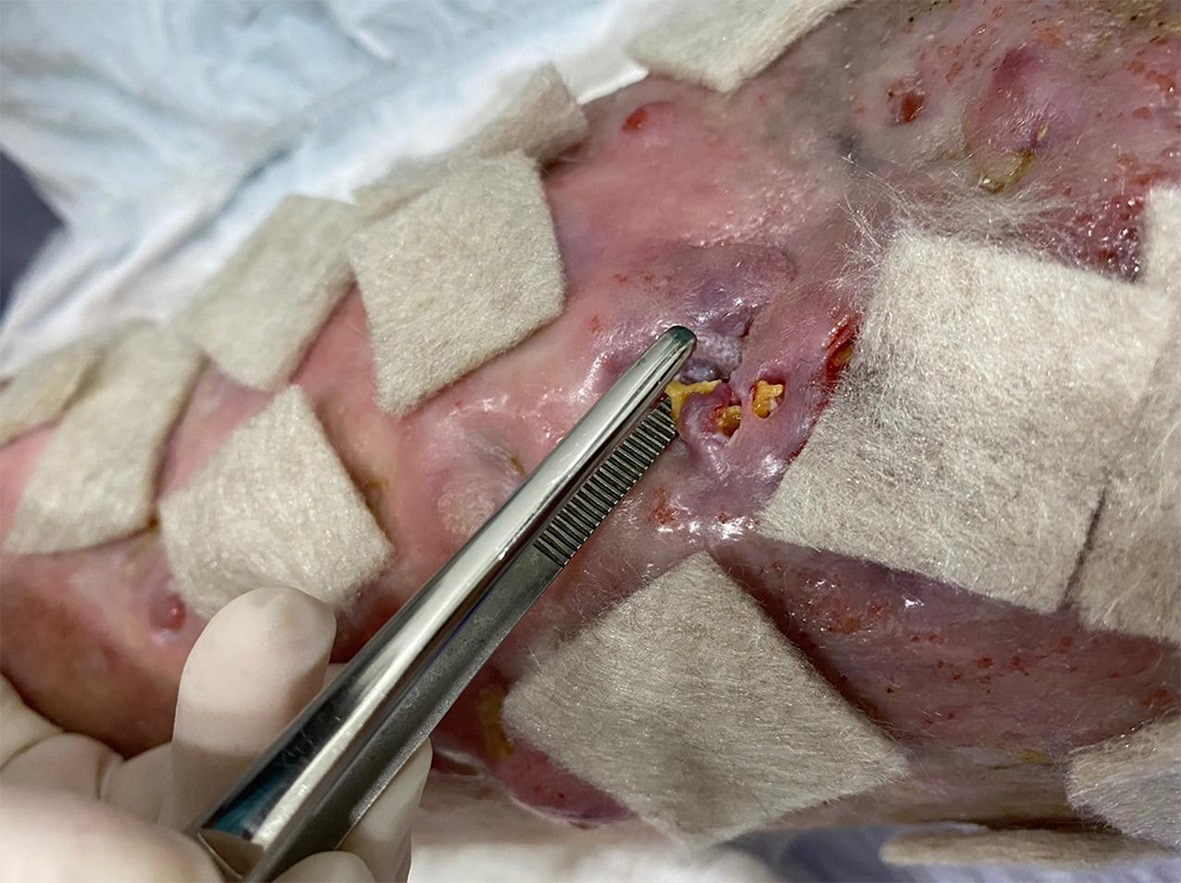
Mechanical extraction of calcifed material.

Treatment of DCC is challenging and the absence of prospective studies compounded by the small number of reports in the literature makes it difficult to evaluate efficacy and safety of the proposed treatment approaches that have been described. Treatment proposals thus range from conservative measures, with regular dressings, removal of material by mechanical extraction (Figure 5) or with the help of drugs, surgical removal, or even ablation, with CO_2_ lasers. However, all of them lack confirmatory evidence.^14^-^18^

## CONCLUSIONS

When associated with CVD, DCC of the lower limbs may be the cause of ulcers refractory to conservative treatment. Management of the condition is challenging and there is no consensus on the best treatment.
