# Supplementary material for: Dystrophic calcinosis cutis in chronic venous disease of the lower limbs
Source: J Vasc Bras. 2022 Sep 2;21:e20210166. doi: 10.1590/1677-5449.202101661 (PMC9477477; doi:10.1590/1677-5449.202101661)
Supplement: Translation

# Translation: Calcinose distrófica cutânea na doença venosa crônica de membros inferiores

**DOI:** 10.1590/1677-5449.202101662

**Authors:** Carolina Heil Arostegui Pacheco, Carmen Lucia Lascasas Porto, Juliana de Miranda Vieira, Ana Leticia de Mattos Milhomens, Rossano Kepler Alvim Fiorelli, Stenio Karlos Alvim Fiorell, Marcos Arêas Marques

**Affiliations:** 1 Universidade do Estado do Rio de Janeiro – UERJ, Hospital Universitário Pedro Ernesto – HUPE, Rio de Janeiro, RJ, Brasil.; 2 Universidade do Estado do Rio de Janeiro – UERJ, Rio de Janeiro, RJ, Brasil.; 3 Universidade Federal do Estado do Rio de Janeiro – UNIRIO, Hospital Universitário Gaffrée e Guinle, Rio de Janeiro, RJ, Brasil.; 4 Universidade Federal do Estado do Rio de Janeiro – UNIRIO, Rio de Janeiro, RJ, Brasil.

**Keywords:** insuficiência venosa, úlcera cutânea, úlcera de perna, úlcera varicosa, calcinose, cicatrização de feridas

## Abstract

As úlceras de membros inferiores, secundárias à doença venosa crônica (DVC), constituem um problema significativo de saúde pública no Brasil e representam cerca de 70% do total dessas úlceras. Apesar dos recentes avanços tecnológicos e das diversas opções terapêuticas utilizadas para essas lesões crônicas, existem diversos fatores que podem estar implicados na resistência ao tratamento. A calcificação distrófica cutânea (CDC) é uma condição rara e frequentemente subdiagnosticada, que, quando associada à DVC, pode estar associada à refratariedade no processo cicatricial. Neste artigo, relatamos um caso de CDC em paciente portador de DVC e discutimos a sua etiologia, fisiopatologia e possíveis opções de tratamento.

## INTRODUÇÃO

A calcinose cutânea (CC) é caracterizada pela deposição anormal de sais de cálcio na pele e tecido celular subcutâneo (TCS), sendo dividida em cinco subtipos: distrófica, metastática, iatrogênica, idiopática e calcifilaxia^1^-^3^.

A calcificação distrófica cutânea (CDC) ocorre em pacientes que não apresentam disfunção sistêmica do metabolismo do cálcio^3^,^4^, podendo ser desencadeada por um dano local, como trauma, infecção ou neoplasia, que leva à degeneração das fibras colágenas e elásticas^5^.

Embora a patogênese da CDC na doença venosa crônica (DVC) ainda não esteja totalmente esclarecida, uma pequena série de casos na literatura atribui o depósito de fosfato de cálcio insolúvel nos tecidos que potencializa e perpetua a reação inflamatória local à estase venosa e ao processo inflamatório crônico cutâneo, que levam a uma dificuldade de cicatrização. Além da DVC, há também relatos de associação da CDC com *diabetes mellitus*, hiperparatireoidismo, doença renal crônica em estágio avançado, doenças autoimunes, doenças do tecido conjuntivo, câncer e infecções cutâneas^1^,^2^,^5^.

O presente caso relata um paciente com úlcera de membro inferior esquerdo, secundária à DVC, com cerca de 24 anos de evolução e refratária ao tratamento devido a associação com CDC.

Este estudo obteve o parecer consubstanciado do Comitê de Ética e Pesquisa do Hospital Universitário Pedro Ernesto (HUPE) da Universidade do Estado do Rio de Janeiro (UERJ), número: 5.174.833 (CAAE: 52753821.2.0000.5259).

## DESCRIÇÃO DO CASO

Paciente de 70 anos de idade, do sexo masculino, portador de hipertensão arterial sistêmica e *diabetes mellitus* tipo I, com índice de massa corpórea maior que 30 kg/m^2^ e DVC. Relatou surgimento de úlcera em membro inferior esquerdo aos 46 anos de idade, com duração de cerca de 20 anos e cicatrização completa por 1 ano nesse período. O paciente apresentou recidiva das ulcerações há cerca de 2 anos, quando foi encaminhado ao Hospital Universitário Pedro Ernesto (HUPE) da Universidade do Estado do Rio de Janeiro (UERJ).

Na história patológica pregressa, relatava erisipela de repetição em membros inferiores e escleroterapia ecoguiada com espuma densa em veia safena magna à esquerda há cerca de 2 anos.

Ao exame físico, observou-se hiperpigmentação “em bota” de terços médios e inferiores de membros inferiores e pele com aspecto de “casca de laranja”, ressecada e com áreas de eczema. Apresentava, também, edema 2+/4+, lipodermatoesclerose, vesículas linfostáticas, lesões múltiplas ulceradas no terço médio distal da perna esquerda, com diâmetro variando de 1,0 a 2,5 cm, com exsudato moderado, sem sinais de infecção secundária e presença de extrusão espontânea de material fragmentado, pétreo, de coloração esbranquiçada (Figura 1). O membro inferior direito não apresentava úlcera. Os pulsos arteriais dos membros inferiores eram amplos, isócronos e isóbaros.

**Figura 1:** Lesões múltiplas ulceradas no terço médio distal da perna esquerda, com diâmetro variando de 1,0 a 2,5 cm, com exsudato moderado, sem sinais de infecção secundária e presença de extrusão espontânea de material fragmentado, pétreo e de coloração esbranquiçada. Foi utilizada cobertura de alginato de cálcio e sódio.

Nos exames laboratoriais solicitados, destacou-se a ausência de alterações dos parâmetros do metabolismo do cálcio (cálcio total: 9,8 mg/dL, fósforo: 4,0 mg/dL, paratormônio: 57 pg/mL, creatinina sérica: 0,9 mg/dL e dosagem de 25 OH vitamina D: 35 ng/mL).

Os testes laboratoriais relacionados à investigação de doenças do tecido conjuntivo não foram reativos, e as culturas para pesquisa de infecção bacteriana e fúngica foram negativas. Foi solicitado o exame histopatológico, que revelou CDC associada a angiodermatite fibrótica difusa.

A radiografia simples do membro inferior esquerdo evidenciava lesões compatíveis com calcificações amorfas em partes moles de face externa de terço médio e inferior da perna esquerda (Figura 2).

**Figura 2:** Radiografia simples do membro inferior esquerdo: lesões compatíveis com calcificações amorfas em partes moles de face externa de terço médio e inferior de pernas.

O ultrassom Doppler colorido (UDC) de membros inferiores revelou refluxo em veia safena magna esquerda, sendo afastados sinais de trombose venosa profunda aguda ou prévia. No modo B, no nível subcutâneo, foram observadas imagens hiperecogênicas que geraram sombra acústica intensa, com áreas de confluência, o que sugere depósito de cálcio no TCS, no terço médio distal da perna na topografia das úlceras (Figuras 3A e 3B).

**Figura 3:** A e B) Ultrassom Doppler colorido de membros inferiores (modo B) com imagens hiperecogênicas que geraram sombra acústica intensa, com áreas de confluência, sugerindo depósito de cálcio no tecido celular subcutâneo, no terço médio distal da perna na topografia das úlceras.

A tomografia computadorizada de membros inferiores evidenciou depósito difuso de cálcio em distribuição concêntrica nos terços médio e distal de ambos os membros inferiores, sem presença nas demais regiões do corpo (Figuras 4A e 4B). A avaliação arterial evidenciou fluxos com morfologia trifásica e velocidades normais.

**Figura 4:** A) Tomografia computadorizada de membros inferiores (cortes transverso e longitudinal) evidenciando depósito difuso de cálcio em distribuição concêntrica nos terços médio e distal de ambos os membros inferiores, sem presença nas demais regiões do corpo.

Como proposta terapêutica, optou-se pelo tratamento conservador, com curativos regulares realizados no ambulatório, com remoção do material pétreo com pinça e uso de cobertura de alginato de cálcio e sódio (Curatec^®^).

## DISCUSSÃO

São cinco os subtipos de CC descritos na literatura médica: metastática, iatrogênica, idiopática, calcifilaxia e distrófica^1^-^3^,^5^.

Na CC metastática, há alteração no metabolismo de cálcio e fósforo, levando a hipercalcemia e/ou hiperfosfatemia; já a CC iatrogênica é secundária à administração intravenosa de gluconato de cálcio ou terapia com fosfato^2^,^6^.

A etiologia da CC idiopática não foi esclarecida, e exemplos dessa condição incluem a calcificação escrotal em homens jovens e nódulos calcificados na cabeça ou extremidades de indivíduos saudáveis, sem qualquer distúrbio metabólico do cálcio ou danos aos tecidos subjacentes^2^,^5^,^6^.

A calcifilaxia é definida como calcificação da camada média de vasos sanguíneos pequenos e médios na derme e TCS em pacientes portadores de doença renal crônica terminal. Clinicamente, a calcifilaxia pode se manifestar com livedo reticular racemoso, que evolui para púrpura retiforme e necrose cutânea^2^,^6^,^7^.

A CDC está relacionada a lesão traumática, química ou infecciosa que leva a depósito anormal de sais de cálcio compostos por hidroxiapatita e fosfato de cálcio amorfo na pele, em pacientes sem disfunção sistêmica do metabolismo do cálcio. Essa deposição provoca inflamação tecidual persistente que, ao final, gera hipóxia e hipovascularização cutânea e induz a liberação de proteínas ligadoras de fosfato, favorecendo a calcificação tecidual. Também estão relacionadas a esse processo as citocinas pró-inflamatórias, como interleucinas 6 e 1 beta e fator de necrose tumoral alfa^2^,^5^,^8^,^9^.

Na CDC, o metabolismo e os níveis séricos de cálcio e fosfato são normais, e, histologicamente, os sais de cálcio são reconhecidos na derme ou na gordura subcutânea^5^. Ainda, o início do processo de calcificação intracelular ocorre em nível mitocondrial das células em apoptose, que perdem a capacidade de autorregulação do cálcio, permitindo o seu influxo em grande quantidade e provocando a falência do metabolismo, necrose e morte celular, sendo o final desse processo o acúmulo de sais de cálcio na forma similar a apatita^2^,^5^.

Histologicamente, os sais de cálcio são reconhecidos como grânulos finos e pequenos depósitos na derme ou depósitos amorfos no tecido subcutâneo. A sua frequência varia na literatura entre 10 e 40% a depender da técnica de avaliação^10^. Lippmann e Goldin^11^ reportaram calcificação no TCS em 10% dos 60 pacientes com DVC avaliados radiograficamente. Outros estudos com radiografia simples evidenciaram que a CDC varia entre 17,5% e 40% nos pacientes com DVC^12^,^13^. Já Takoro et al.^4^ relacionaram a prevalência de CDC latente na DVC maior que 65% em comparação com a população geral, sugerindo que a detecção precoce da calcinose latente com radiografia simples pode ser útil na tentativa de inibir a sua progressão.

Este estudo tem como limitação a perda de seguimento do paciente durante o seu acompanhamento.

## TRATAMENTO

O tratamento da CDC é desafiador, e a pequena série de relatos na literatura, associada à ausência de estudos prospectivos, dificulta ainda mais a avaliação da eficácia e da segurança das propostas de abordagem terapêuticas descritas. Nesse contexto, existem propostas de tratamento, envolvendo desde medidas conservadoras, com curativos regulares, remoção do material com extração mecânica (Figura 5) ou com auxílio de medicamentos, remoção cirúrgica ou até opções ablativas, como *laser* de CO_2_. No entanto, todas carecem de comprovação^14^-^18^.

**Figura 5:** Extração mecânica do material calcificado.

## CONCLUSÃO

A CDC, quando associada à DVC dos membros inferiores, pode ser causa de úlceras refratárias ao tratamento conservador. O manejo dessa condição é desafiador, e não há consenso sobre o tratamento mais adequado.
